# Impacts of climate change on dogsledding recreation and tourism in Arctic Sweden

**DOI:** 10.1007/s00484-023-02542-z

**Published:** 2023-09-07

**Authors:** Robert O. Nilsson, O. Cenk Demiroglu

**Affiliations:** 1 Department of Geography, Umeå University, Umeå, Sweden; 2 Arctic Centre, Umeå University, Umeå, Sweden

**Keywords:** Climate change, Dogsledding, Tourism, GIS, Sweden, Arctic

## Abstract

The range of Arctic tourism supply is continuously increasing with a variety of tourism products on offer. However, climate change is becoming a more prominent issue threatening the operations of tourism businesses and the livelihood of some tourism actors, such as dogsledders. This article aims to fill the descriptive research gap that exists regarding the dependency on the physical environment, climate, and weather for dogsledding activities. This is achieved by studying how climate change may threaten possible climate and weather thresholds for these activities, and how climate change may affect the future opportunities for dogsledding in northern Sweden. The study is based on interviews with dogsledders in Arctic Sweden and climate projections from the Swedish Meteorological and Hydrological Institute (SMHI). The results demonstrate the following thresholds for dogsledding activities: (1) dogsledding requires 10–20 cm of packed snow and/or solid ice on bodies of water, (2) above 15 °C is too hot for dogs to pull (wheeled) sledges, (3) cold weather thresholds are determined by visitors’ preferences and are not considered a problem for dogsledders or dogs, and (4) rain can cancel tours for all dogsledders, and strong wind can cancel tours for dogsledders located in the mountain regions. Finally, extreme events such as heatwaves, storms, thunderstorms, forest fires, heavy rain, floods, and more rapid weather changes have already affected some dogsledders. These necessary thresholds for dogsledding activities could already be jeopardized for the southern and coastal locations of Arctic Sweden. In addition, the climate projections from SMHI show that warmer days and more precipitation in the form of rain will become more common in the future, especially in the absence of global mitigation measures. However, further research on vulnerability/resilience and adaption strategies for dogsledding activities is necessary to truly understand the impact of climate change.

## Introduction

Understanding the factors behind destinations’ attractiveness has always been an important aspect of tourism research (Gearing et al. 1974). Previous studies have shown that destinations’ physical attributes such as landscape, climate, and other natural resources are essential parts of understanding tourist flows and destinations’ advantages and disadvantages (Gössling and Hall 2006). Hence, understanding the dependency on the physical environment, climate, and weather from a supply-side perspective is essential. Thus, this article aims to provide descriptive knowledge about dogsledders’ dependency on their natural environment. Existing research identifies factors that have been found to influence tourists’ perceptions and expectations of destinations (Lohmann and Kaim 1999), and how they affect the tourists’ experiences at the destinations (Moreno 2010). For example, research has shown that climate and weather are relevant factors in tourists’ decision-making process and choice of tourist destinations (Hamilton and Lau 2005). Destinations’ physical attributes such as accessibility to the sea, beaches, certain types of landscapes, and weather act as various push and pull factors for different groups of tourists based on the tourists’ origins (Kozak 2002), including seasonal weather at both the tourist generating regions and the potential destinations (Maddison 2001). Similarly, weather factors such as temperature and precipitation have been shown to influence tourists’ behaviors and activities at their chosen destinations (Moreno et al. 2009). Research has shown that tourism and meteorological organizations around the world acknowledge that climate and weather are of major importance for tourism in their respective countries (cf. Wall and Badke 1994).

There has been an increased call in tourism research for understanding the influence climate and weather have on a destination’s attractiveness, the possibilities of various tourism activities, the tourists’ choices of destinations, and their behavior and experiences at the destination. For these purposes, climate indexing has been a prominent methodology. The pioneering (and one of the most cited sources to date) of such indices is the tourism climatic index (TCI) by Mieczkowski (1985). Even though the study introduced indices for climate and weather and highlighted the importance of these types of factors for tourism already in the 1980s, Mieczkowski’s work has since then been criticized and seen as limited (cf. Scott et al. 2016). This has led to several modifications and improvements of different climate indices such as the beach climate index (BCI) (Morgan et al. 2000), the climate index for tourism (CIT) (de Freitas et al. 2008), the modified climate index for tourism (MCIT) (Yu et al. 2009), the holiday climate index (HCI) (Scott et al. 2016; Rutty et al. 2020; Demiroglu et al. 2020a), and the ski climate index (SCI) (Demiroglu et al. 2021). The general idea for all these indices has been to develop useful tools to measure, as objectively as possible, various climate and weather factors, such as precipitation, thermal comfort, wind, and other variables, to determine the climatic attractiveness of different types of tourism destinations (de Freitas 2003). These indices are also important to gain a better understanding of destinations’ livelihoods and their future adaption possibilities due to climate change (Demiroglu et al. 2021), which also requires the adoption of indices to different forms of tourism, as different types of tourism activities may depend on distinct climate and weather thresholds (Dubois et al. 2016).

### Climate and imaginary change in tourism

Ross (1993) states that for tourism, varied physical environments are one of the major factors contributing to a positive experience of a destination and willingness to recommend it to others. Having access to nature and natural attributes for nature-based activities is often seen as an essential part of the tourism supply (Margaryan 2018). Natural environments are in some places an important resource for tourism branding strategies (Wall Reinius and Fredman 2007), resulting in tourism not only having physical impacts on places but also influencing the construction and promotion of various images of places (Bryant 2000). Hunt (1975) argues that these imaginary projections of destinations might have a similar impact on the perception of a destination to accessibility, physical facilities, and intervening opportunities. This raises concern about climate change, changing weather, various thresholds, and the impact on tourism destinations. As destination organizations and stakeholders produce, sell, and market certain climate and weather imaginations to promote a specific perception, it may become problematic if the environment changes and no longer correspond with what is being promoted (Hall 2008). For Arctic winter tourism, descriptions of snow, ice, cold, darkness, northern lights, and other environment-related words and images are often utilized in branding, marketing, and promotion of places, tourism destinations, tourism business names, tourism products, services, and more (e.g., Jacobsen 1997; Lucarelli and Heldt Cassel 2019; Marjavaara et al. 2022; Viken and Granås, 2014). In the Arctic region, this type of “Arctification process” where winter and Arctic images are practiced has led to benefits such as new economic and social relations (Müller and Viken 2017). Some studies (Hall 2014; Demiroglu et al. 2020b), on the other hand, have shown that climate change will result in both opportunities and threats for the Arctic winter images and tourism.

Tourism destinations, businesses, and products that are dependent on certain climate and weather thresholds are more sensitive to and more affected by climate change (Saarinen and Tervo 2006; Scott et al. 2012; Kaján, 2014). According to the IPCC, the tourism sector has already started to suffer from economic damages and livelihood failure due to climate change (high confidence), and winter tourism activities such as ski tourism in many places have already suffered from a lack of snow reliability and increased snow-making demand (IPCC 2022). Ski tourism is heavily reliant on certain climates and weather thresholds (Demiroglu et al. 2021). This has led ski resorts to consider various adaptation strategies due to climate change (Bicknell and McManus 2006) and strive for competitive advantage in the changing climate (Steiger and Mayer 2008). Previous studies on ski tourism have investigated the importance of tourism attitudes to climate change and the possibilities, effects, and prevalence of adaption strategies, for example, snow storing and snowmaking (cf. Hennessy et al. 2003; Pickering et al. 2009; Scott et al. 2003; Scott and McBoyle 2007; Whetton et al. 1996). Hence, climate dependency and thresholds, climate change, and adaption strategies for ski tourism have been researched in several studies (Steiger et al. 2019). Yet, as stated by Tervo (2008), the thresholds and climate vulnerabilities of other winter tourism activities, such as dogsledding, and their adaption possibilities have not been studied, even though these activities are similarly dependent on certain winter climates and weather thresholds.

### Dogsledding and climate change

According to Hall et al. (2009), dogsledding is an important part of the winter tourism supply and a clear example of how the Arctic, snow, cold, and other winter aspects are used by tourism businesses when promoting activities to the market. Yet, few studies have researched the effect of climate change on dogsledding businesses. Hence, this article aims to fill the descriptive research gap that exists about the dependency on the physical environment, climate, and weather for dogsledding activities to enrich the previous studies which include climate vulnerability (Tervo 2008 in Finland), climate impact assessment (Schrot et al. 2019 in Greenland), adaptation (Brouder and Lundmark 2011 in Sweden; Tiller and Richards 2018 in Norway), and tourists’ perceptions of climate change (Tervo-Kankare et al. 2013 in Finland) for winter tourism activities, and in particular, dogsledding. All studies are presented below to provide information on the limited knowledge that exists on the topic.

Tiller and Richards (2018) researched stakeholders in different industries and their willingness to accept and also adapt to the changing marine environments in Northern Norway. The authors found that northern lights tours were important for the tourism sector, and today, these tourism tours are mainly conducted with the use of dogsleds or snowmobiles. However, if climate change would result in snow disappearing in the region, dogsledders’ only option would have to be to adapt their products and tours by using all-terrain vehicles instead of their dogs and snowmobiles. Furthermore, seven different types of winter tourism activities in West Greenland including dogsledding tours were studied by Schrot et al. (2019). Their study showed that climate change had reduced the snow cover thickness, ice sheet in bays, and general snow cover at the coast of the region, leaving dogsledders with trails that were seen as unsafe and unusable for dogsledding activities. Climate change also led to fewer tours and cancellations, forcing dogsledders into adapting their tours to “climate change tours.” These dogsledding tours were created for tourists to experience melting ice/snow before the Arctic landscape supposedly disappears (ibid). These findings provide a good example of what Lemelin et al. (2010) describe as “last chance tourism,” i.e., experiencing something before it disappears, often related to climate change.

Tervo-Kankare et al. (2013) found that dogsledding activities in Santa Claus Village in Rovaniemi, Finland, were of great importance among tourists. The authors concluded that only three activities were perceived as more important for tourists visiting the destination: these were (1) visiting Santa Claus, (2) a visit to the Arctic Circle, and (3) reindeer activities. Some notable less important activities included snowmobiling, seeing the northern lights and downhill skiing, and demonstrating that dogsledding is a highly ranked winter tourism activity among tourists to one of the most famous winter tourism destinations in the Arctic. In addition, Tervo-Kankare et al. (2013) state that most tourists that had visited Santa Claus Village and the Arctic Circle had also partaken in dogsledding and that this type of activity could not be performed without proper snow cover.

Brouder and Lundmark (2011) studied different types of tourism businesses in northern Sweden and demonstrated that the entrepreneurs of these businesses had different perspectives on climate change which depended on two major factors. Firstly, in terms of location, tourism entrepreneurs in the coastal regions experienced greater changes in the climate compared to the ones in the inland and mountain regions. Secondly, in terms of the type of tourism product, tourism entrepreneurs who managed venue-based attractions such as ski resorts were more concerned about climate change compared to tourism entrepreneurs with activity-based businesses that were more flexible to move their activities elsewhere, for example, dogsledding businesses. Furthermore, Brouder and Lundmark (2011) stated that future qualitative studies investigating tourism businesses and climate change in northern Sweden are necessary.

Lastly, Tervo (2008) is the only study which focuses on the climate vulnerability of various winter tourism activities that specifically included dogsledding. The author found that dogsledders’ minimum requirements for ice thickness were 15–20 cm and snow depth had to be at least 11–20 cm. This 20-cm threshold was also found in an interview in Greenland by Schrot et al. (2019). However, it was not specified if the measurements refer to the snow depth in the packed and prepared trails or the natural snow cover in either of these studies. Moreover, Tervo (2008) reported that weather-related cancellations occurred due to a lack of snow, high temperatures, and rain, again, the same results for found in the Greenlandic study by Schrot et al. (2019) that also highlights the negative consequences of increasing rainfall. Tours could also be cancelled due to international visitors’ inexperience with cold temperatures, yet details or measurements of what temperatures that might be were not specified. Adaption strategies were included in the study, and it was found that some dogsledders used snow cannons, stored snow, and/or transferred their activities if needed. Finally, Tervo (2008) concluded that rain and temperatures above 0 °C were the most negative and critical factors that would hinder all types of all winter activities studied. Dogsledding and snowmobiling were found to be the most vulnerable of all winter activities if the precipitation of snow decreased. In addition to these articles, a dissertation by Äijälä (2022) on dogsledding-human-place relations in tourism highlights the importance of dogsledding as a tourism activity in northern Finland. Furthermore, it is stated that dogsled training starts when the temperature is below 15 °C, to avoid overheating the dogs (ibid).

The presented articles demonstrate the lack of knowledge of the impact of climate change on dogsledding activities. Tervo (2008) came one step closer to building a better understanding of dogsledders’ relation to climate and provided evidence of the heterogeneity of different winter tourism businesses and their dependence on climate and weather thresholds. The article calls for new research on each type of winter tourism activity and their unique requirements related to climate and weather thresholds. Hence, this article aims to fill the descriptive research gap that exists about the dependency on the physical environment, climate, and weather for dogsledding activities, by studying the climate and weather thresholds and how climate change may impact dogsledding activities. This was achieved by mapping the climate and weather thresholds that dogsledding activities have according to the dogsledders themselves and how climate change could affect their ability to conduct these activities.

## Materials and methods

This study adopted a mixed method with quantitative and qualitative approaches. The main workflow included (1) the identification and mapping of the dogsledding businesses in Northern Sweden, (2) interviews with the dogsledders about climate and weather dependency, and (3) visualizing and matching the findings that emerged from the interviews with future climate change projections using data from the Swedish Meteorological and Hydrological Institute (SMHI) and present the data using geographical information systems (GIS).

### Mapping dogsledders in Northern Sweden

The spatial delimitation for this study was set to Sweden’s two northernmost counties, Norrbotten and Västerbotten. These counties were selected as they are defined as Sweden’s Arctic region in the “Swedish Strategy for the Arctic Region” produced by the Swedish Government (Regeringskansliet 2020). Dogsledding businesses in Sweden do not have an industry association that could have been contacted, nor do dogsledding businesses have SNI codes, which are used in Sweden to attribute businesses to one or more industries (Statistic Sweden 2022). To find the dogsledding businesses located within the region, manual research had to be performed and was conducted in four steps. In the first step, relevant national organizations including the Swedish Agency for Economic and Regional Growth (Tillväxtverket), Statistic Sweden (Statistiska Central Byrån), and the Swedish Tax Office (Skatteverket) and tourism-specific organizations such as the National Organization for the Swedish Hospitality Industry (Visita), the Swedish National Organisation for Tourism and Travel Information (Visit Sweden), the Swedish Tourism Association (Svenska Turistföreningen), and the Swedish Nature Tourism Business Association (Naturturismföretagen) were contacted. These organizations could only provide information about a few dogsledding businesses. However, the organizations did provide contact details for all regional tourism organizations and destination management organizations (DMOs) in the study area. In the second step, each municipality office, county tourism organization, and DMO were contacted in both Norrbotten and Västerbotten to find as many dogsledding businesses as possible. The approach resulted in tracking down most of the dogsledding businesses. Thirdly, adopting the snowball sampling method when speaking to the dogsledders and asking them about additional dogsledders they might know of resulted in discovering a handful of dogsledding businesses. These businesses were previously unknown, partly because these dogsledders were about to shut down mainly due to the effects of the pandemic. Lastly, the entire process was constantly supplemented with online searches by the researchers for any other dogsledding businesses in the region that might have been missed in the other steps.

**Fig. 1:**
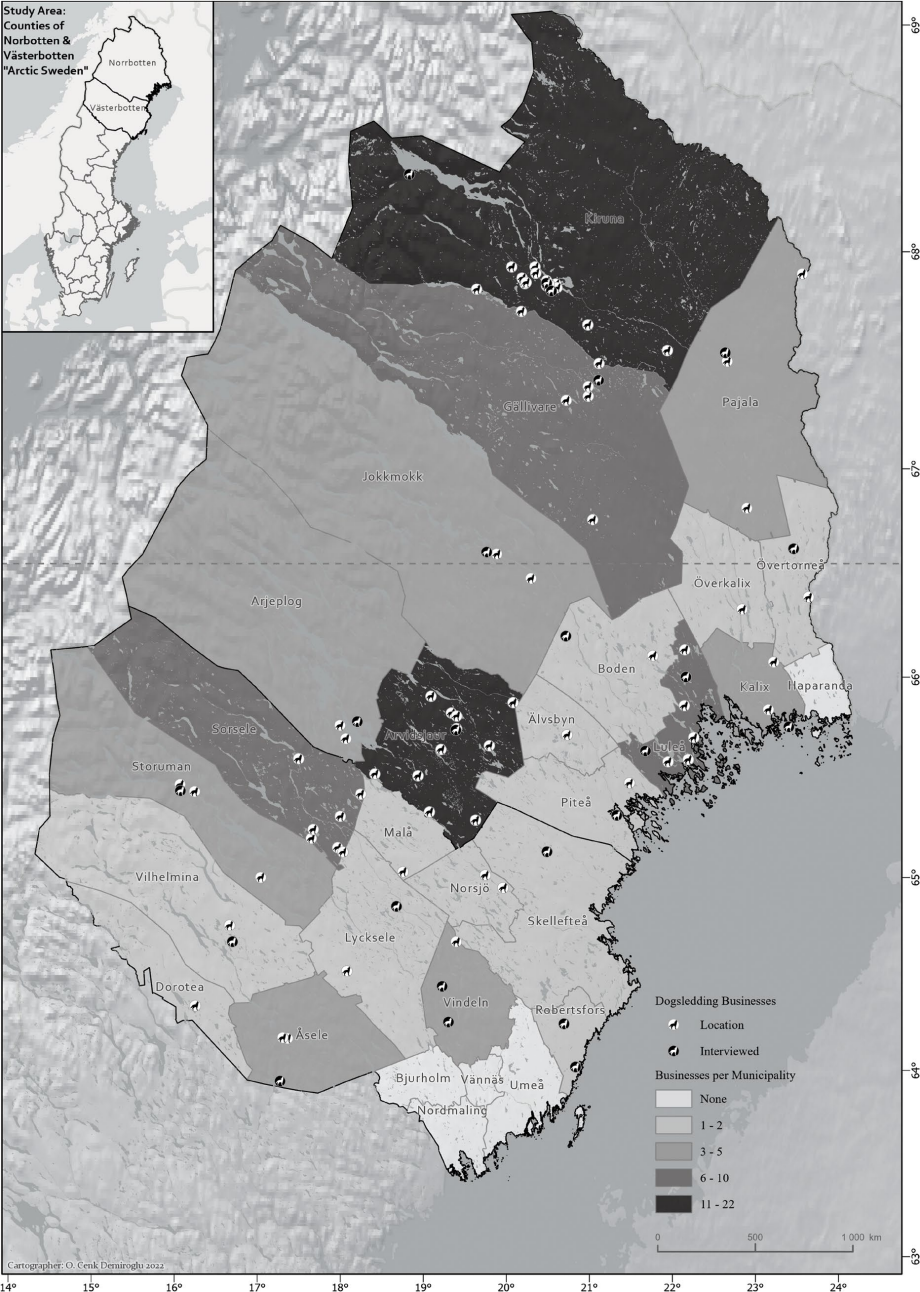
Dogsledding businesses in Arctic Sweden

Eventually, 97 active dogsledding businesses were identified within the region and mapped according to their addresses and online information (Fig. 1), assuming that their operations take place close to their business addresses. Sixty-eight businesses (70%) are in Norrbotten and 29 (30%) in Västerbotten. Kiruna is the municipality with the most businesses (*n*: 22), with a major cluster surrounding Jukkasjärvi (a village east of Kiruna), where the world-renowned Ice Hotel is situated. Arvidsjaur is the second most populated (*n*: 12) municipality, reflecting the general pattern of the dogsledding businesses’ locations in the sparsely populated inland. While the businesses seem more or less dispersed throughout the study area, the more mountainous regions to the west, with some medium and large ski resorts as the major attractions, do not offer any dogsledding activity, as determined by the researchers, except maybe in Abisko in the west of Kiruna municipality. Besides the inland supply, some coastal municipalities also have dogsledding businesses, especially at the higher latitudes closer to the Arctic Circle and with relatively strong population centers such as Luleå. Some of the business locations at these coastal municipalities are in very close and sometimes immediate proximities to the marine coastline. Such water proximity is indeed often the case with these businesses as almost all inland activity areas are also by, or very close to, rivers or lakes. Among the 29 municipalities of the study area, only Haparanda in Norrbotten and the southernmost municipalities in Västerbotten lacked dogsledding businesses. However, it should be noted that Umeå is the main population center of the region and thereby acts as both a main demand source and a hub/gateway to connect more national and international visitors to these businesses in Arctic Sweden, especially Västerbotten.

### Interviews with dogsledders in Arctic Sweden about weather and climate

To better understand dogsledding activities in the case study area in terms of climate and weather, an interview guide was initially created based on the relevant literature (Tervo 2008; Brouder and Lundmark 2011; Tervo-Kankare et al. 2013; Tiller and Richards 2018; Schrot et al. 2019). This semi-structured, first version included a direct section on weather and climate that seeks to disclose the critical variables and any extreme events relevant to these businesses. After a pilot interview with one of the businesses, it was brought to the authors’ attention that they had a maximum temperature for their dogs. Accordingly, the interview guide was updated to include discussions of any thresholds not only for the visitors but also for the dogsledders themselves and/or their dogs. These changes proved beneficial, as throughout the interviews, the informants did not have a particular preference regarding the ideal conditions of climate or weather but had clear thresholds for the climate and weather to be able to conduct dogsledding activities. This modification also justified the use of interviews with the dogsledders instead of visitor surveys for data collection, as the dogsledders themselves should be the best sources to reflect an overall picture from both supply and demand perspectives. The interview guide included an initial section where the dogsledders introduced themselves and their businesses in terms of the informants’ position, nationality, motivation for starting the business, the start date of the business and its history, what they offered, possible staff, number of dogs and dog breeds, and number of visitors. The climate-related questions included seasonal length and the range, thresholds, and effects of the following types of climate and weather factors: snow and ice cover, temperature, precipitation (snow and rain), wind, and visibility (fog, mist and cloudiness). Lastly, the informants were asked to rank these in order of importance for dogsledding activities and describe any extreme weather event they had experienced and/or believed had increased during their time with the business.

The interview guide was accompanied by an introductory note on the study with preliminary visualizations of the critical future snow cover variable to grab more enthusiasm for participation. The study followed the generally accepted ethical considerations for social science by the Ethics Review Authority (Etikprövningsmyndigheten 2022) and the Swedish Research Council (Vetenskapsrådet 2022), and a consent form was also included to clarify the voluntary positions of the informants and to ask their permissions for direct and non-anonymous quotations and to record the interviews. All 97 identified dogsledders were contacted by phone and asked to participate in the study. The response rate was at 24% (n: 23), with the main reasons for the decline being lack of time or interest. The 23 informants (Fig. 1) reflected a relatively representative sample of the spatial distribution of the businesses in the region. The sample include *n*: 23 businesses, *n*: 14 (60%) of which were located in Norrbotten and *n*:9 (40%) located in Västerbotten of the total population include which was *N*: 68 businesses (70%) in Norrbotten and *N*: 29 (30%) in Västerbotten. There is also a relatively fair spatial distribution among the informant’s location in each municipality and the inland, mountain, and coastal zones. Each of the informants was interviewed by phone, in Swedish or English depending on the capability and preference of the informants. The interviews were carried out between the 7th of June and the 25th of July in 2022 and each lasted approximately 45 to 90 min. Twenty-one of *n:* 23 interviews were recorded, and the remaining two were carefully followed by taking notes. The final corpus was summarized per variable with a focus on climate and weather thresholds.

### Profiles of dogsledding businesses in Northern Sweden

Twenty-three interviews were conducted out of 97 active dogsledding businesses in the region. All informants were owners of the dogsledding businesses, and the majority were owned by at least one person of Swedish background (*n*: 17); see Table 1. The people with non-Swedish national backgrounds were from other European countries and North America, reflecting the composition of the main population where 28 of the 97 dogsledders were identified by the researchers to have at least one owner being of non-Swedish background. All informants described that dogs, dogsledding, nature, and the lifestyle were their main interests (*n*: 23). However, some informants specified that the main reason for starting the business was for tourism purposes (*n*: 3), and two others explained that they did tourism to finance themselves for dogsled racing. All businesses were micro-businesses, with a staff consisting of 1–6 (mean: 2.4) people, the owner(s) included. Some business was run solely by the business owner(s) (*n*: 12) and some had additional staff (*n*: 11). The staff consisted of either employees or volunteers and in some cases were hired on a seasonal basis; see Table 1.

**Table 1:** Business information on dogsledders in Arctic Sweden (1)

	Frequency (*n*)	Percentage (%)
Informants interviewed		
In Norrbotten	14	60.87%
In Västerbotten	9	39.13%
Nationalities		
Swedish background	12	52.17%
Swedish and non-Swedish background	5	21.74%
Non-Swedish background	6	26.08%
Prime motivation for starting		
Tourism	3	13.04%
Dogsled racing	2	8.69%
Dogs, dogsledding, nature, lifestyle	18	78.26%
Type of staff		
Owners only	12	52.17%
Employees	8	34.78%
Volunteers	3	13.04%

The average annual visitor volumes ranged from 40 to 50 visitors/season to more than 2000; see Table 2. The variation was dependent on the number of dogs, i.e., the more dogs, the more visitors could go dogsledding at the same time, and if the businesses offered accommodations and had the same visitors over a longer time. The number of dogs per business ranged from 16 to 303 (median: 34), where Alaskan Huskies and Siberian Huskies were the most common breeds, followed by Greenland Dogs, Samoyeds, and a variety of other breeds that were not used for dogsledding.^1^

**Table 2:** Business information on dogsledders in Arctic Sweden (2)

	Frequency (*n*)	Percentage (%)
Number of guests		
40 to 150	6	26.08%
151 to 350	6	26.08%
351 to 800	6	26.08%
> 800	3	13.04%
Did not want to disclose	2	8.69%
Number of dogs		
10 to 20	5	21.74%
21 to 30	5	21.74%
31 to 40	6	26.08%
41 to 55	5	21.74%
> 55	2	8.69%
Dog breeds		
Siberian Husky	7	30.43%
Alaskan Husky	11	47.82%
Siberian Husky, Alaskan Husky, Samoyed and/or Greenland dogs	5	21.74%

**Fig. 2:**
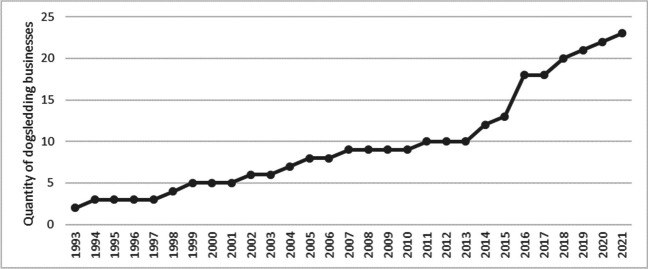
Number of active dogsledding businesses in Arctic Sweden (interviewed)

Last but not least, these businesses, at least in the example of this sample set, indicated that dogsledding recreation and tourism in Northern Sweden are a fairly new trend. Only five of the businesses in the sample existed in the 1990s, doubling by 2011 to 10 and doubling that by 2019 to just exceed 20 (Fig. 2).

### Climate change projections and GIS

Matching the findings that emerged from the interviews with data on the future climate of Northern Sweden required a review of publicly available datasets for this purpose. While relevant tailor-made tourism-climate datasets and applications (Morin et al. 2021; Giannakopoulos et al. 2022) were openly available from the Copernicus Climate Change Service (C3S) of the European Centre for Medium-Range Weather Forecasts (ECMWF), these were aggregated at the NUTS (nomenclature of territorial units for statistics) level 3, thus providing values only for the rather coarse county level in Sweden. Instead, the less tourism-specific but more locally adjusted and refined Climate Index Database for Sweden (SCID 4.0) produced by the Swedish Meteorological and Hydrological Institute (SMHI 2014) was used to visualize and analyze the changing climates of dogsledding recreation and tourism in Northern Sweden. SCID 4.0 bears various meteorological and hydrological indices (see SMHI 2015 for a full list) based on modelling outputs. The model chain (see Sjökvist et al. 2015 for a comprehensive overview) departs from an ensemble of the nine global climate models (GCMs) of the CORDEX (coordinated regional climate downscaling experiment). It is dynamically downscaled by the SMHI’s regional climate model RCA4 from a grid resolution of 100–300 to 50 km for the two greenhouse gases (GHG) concentration trajectories of the Intergovernmental Panel on Climate Change (IPCC), i.e., the mitigation-oriented RCP4.5, and the fossil-intensive RCP8.5 scenarios. A critical final step in the model chain before establishing index datasets is the use of distribution based scaling (DBS) which helps adjust the systematic model errors according to actual field observations (Sjökvist et al. 2015), scaling the meteorological index results further down to a 4-km resolution following the interpolated grids of the field stations. The hydrological indices, on the other hand, could only be further downscaled to a resolution of 21 km on average, but with very heterogeneous polygons according to the catchment areas of the semi-distributed hydrological model HBVSv (Hydrologiska Byrån Vattenbalansavdelningen—Sverige) (Sjökvist et al. 2015). These spatially refined final results can then be retrieved within a file geodatabase that contains vector-format shapefiles for each index-pathway-statistic-unit combination (SMHI 2014). For this study, those indices that were most relevant to the findings that emerged from the interviews and distinguished along different pathways (RCP4.5, RCP8.5) and periods (1991–2013 for the present, 2021–2050 for the near future, 2069–2098 for the far future) and the robust ensemble mean values were utilized for geo-visualization and further spatiotemporal analyses via the software ArcGIS Pro 3.0.2. Regarding projections on extreme events such as floods and fires, results of SCID-based reports (Sjökvist et al. 2015; 2016) were also referred to.

## Empirical findings

This section is presented in two parts, the weather and climate thresholds deemed important by these businesses, and the scenarios showing the future of these thresholds in a changing climate.

### Climatic thresholds for dogsledding recreation and tourism in Northern Sweden

In most cases, there were high levels of agreement among the informants regarding the climatic thresholds of dogsledding in Northern Sweden, but the seasons varied between the businesses; see Table 3. All informants stated that at the beginning of the season, they wanted to start sooner if possible, and at the end of the season, they wanted to stop later if possible to extend the winter season. The conventional season start could be as early as mid-November (*n*: 3), or already in early December around St. Lucia Day (*n*: 6). The early starts were especially seen in the far north, but most commonly, the start of the seasons was around the Christmas-New Year’s Day week (*n*: 12). The latest start in mid-January was a business located in a relatively coastal and southern zone; see Table 3. The season would mostly last till the Easter break around early to mid-April (*n*: 15), with some inland and mountainous businesses aiming for the end of April and early May (*n*: 2). Some businesses offered dogsledding on dryland using wheeled carts or quad bikes for guests to book when the temperatures were still low to not overheat the dogs (*n*: 6). However, only one dogsledder specified a period for when it was used, which was August to October and mid-May to mid-July to extend their season (*n*: 1). The seasons depended on three main climatic factors: snow depth, ice thickness and temperature; see Table 3. The majority of informants (*n*: 20) stated that 10–20 cm of snow depth was required for the packed trails and thus required more snow for extremely uneven terrain. That argument was specified by two informants who said that they go through very uneven terrain and wanted 30–50 cm in their packed trails. The last informant used trial and error to see if it worked. In cases where sledding also took place on frozen lakes, rivers, or the sea, a solid (freezing from the bottom to the top) ice layer with a minimum thickness of 10–20 cm (*n*: 18) was sought, even though one informant said that 5–6 cm was sufficient. Another informant explained that they would try the ice to see if it was strong enough, and finally, the remaining three informants did not go on the ice; see Table 3. Some informants explained that they drilled and measured the ice before going on it (*n*: 3), and remaining dogsledders that had tours on ice tested it with snowmobiles or sledges without guests (*n*: 17).

**Table 3:** Climate and weather threshold for dogsledders in Arctic Sweden (1)

	Frequency (*n*)	Percentage (%)
Start of season		
Mid-November to end-November	3	13.04%
Beginning of December to mid-December	6	26.08%
Mid-December to end-December	12	52.17%
Beginning of January to mid-January	2	8.69%
End of season		
Mid-March to end-March	7	30.43%
Beginning of April to mid-April	4	17.39%
Mid-April to end-April	11	47.82%
Beginning of May to mid-May	1	4.34%
Snow depth (in packed trails)		
10 to 20 cm	20	86.96%
30 to 40 cm	2	8.69%
Trial and error	1	4.34%
Ice thickness (solid ice)		
10 to 20 cm	18	78.26%
20 to 30 cm	2	8.69%
Trial and error	1	4.34%
Does not go on the ice	3	13.04%

In regard to temperatures, the cold thresholds were based on the visitor’s preferences, and the warm thresholds were for the safety of the dogs; see Table 4. All informants (*n*: 23) were clear that the cold did not hinder the dogsledders or dogs in any way, shape, or form. The cold temperatures only required more clothing for the visitors and dogsledders, and if necessary shoes and covers for the dogs. A small majority of the businesses did not have a specified cold limit at all (*n*: 12). Those who did have between − 25 and − 41 °C (*n*: 11) and these thresholds were based on previous visitors’ experiences. However, informants from both groups said that it was up to the visitors if they wanted to go or not. Some specified that if children participated in the tours, it was up to the children to decide when it was too cold to go, or if they wanted to go back (*n*: 3). All informants (*n*: 23) stated that the warm weather threshold was for the dogs’ safety and was for the majority between 10 and 15 °C (*n*: 21) with two exceptions 5 °C (*n*: 1) and 0 °C (*n*: 1); see Table 4. The results did not show any correlations between the dog breeds and the maximum warm temperature. The informants explained that these dog breeds have a normal lifespan of 10–12 years and have used the most suitable dogs for the climate when breeding. Many of the dogsledders had bought their dogs from other dogsledders in the region but not all of them wanted to disclose any information about it. The temperatures in the region do not (yet) get to temperatures that are problematic for the dogs as long as they are not working, i.e., pulling sledges. Humidity was mentioned as the most crucial factor that influenced the warm temperature threshold, as it could make the weather feel warmer (*n*: 18). One informant in the coastal region and one in the mountain region even specified the danger of high humidity for dogs in general but said that they were not affected by this since the humidity in their respective locations was low. The other two factors influencing the warm weather threshold were access to bodies of water to cool down the dogs and/or snow on the ground as dogs sweat through their paws which helps them keep cool. This was also seen as two important factors that influence the length of the season (*n*: 7). All informants stated that warm temperature was for the safety of the dogs and was the most crucial threshold. If the dogs were too “run warm,” they would have problems with overheating for the rest of their lives (*n*: 23).

**Table 4:** Climate and weather thresholds for dogsledders in Arctic Sweden (2

	Frequency (*n*)	Percentage (%)
Temperature—cold limits (threshold for guests)		
− 25 to − 29 °C	2	8.69%
− 30 to − 35 °C	6	26.08%
− 36 to − 40 °C	2	8.69%
− 41 °C <	1	4.34%
No limit, up to guests	12	52.17%
Temperature—warm limits (thresholds for dogs)		
Maximum 0 °C	1	4.34%
Maximum 5 °C	1	4.34%
Maximum 10 to 15 °C	21	91.30%
Precipitation (snow and rain)		
Problematic	7	30.43%
Not problematic	16	69.56%
Wind (speed and direction)		
Problematic	16	69.56%
Not problematic	7	30.43%
Visibility (fog, mist, cloudiness)		
Good visibility	9	39.13%
A mix of different visibility conditions	5	21.74%
No preference	9	39.13%

The other climate factors that were studied were as follows: precipitation, wind, and visibility (fog, mist, cloudiness); see Table 4. Rain was considered by the vast majority of the informants (*n*: 22) as the only crucial weather factor and the most problematic of these types of weather as it could lead to cancellations. The wind was more problematic for one informant in the mountain region as it was described as being potentially dangerous if the wind became too strong. Two informants did mention that rain could be positive if it freezes overnight and stabilizes the trails. Precipitation as snow was not considered a problem on its own (*n*: 23). Yet, in combination with wind, snow could become problematic either due to the effects, such as drifting snow over existing trails (*n*: 5), fallen trees (*n*: 1), tip sledges (*n*: 2), or have a negative effect on visitors’ experiences (*n*: 2). It was also noted that the wind direction (from the north and coast, i.e., cold winds) played a negative role for the comfort of the visitors (*n*: 2). Cold winds in the warmer months of the year were seen as positive to cool down the dogs (*n*: 3). Overall, the informants did not consider the wind as problematic due to the protection in their usual forest trails (*n*: 16). The visibility was not considered a problem for the informants (*n*: 23). However, it was specified that visitors would prefer a more cloud-free experience (*n*: 10), especially when aurora watching (*n*: 1). One informant did say that it could be a negative factor if visitors wanted to take pictures but would not consider it a problem. Some informants argued that they preferred having a mix of weather factors during the same tour for visitors to experience different weather types; see Table 4. Two informants stated that the experience depended on how the dogsledders built up the expectations and that any weather could be “magical”; it was up to the dogsledders to convince the visitors of it. Going through the fog was described as a “magical experience” in itself without having to “sell it” (*n*: 2), and some declared that since they had visitors for several days, they could choose to go in or avoid such weather depending on the visitor’s preferences (*n*: 2).

The informants were asked to rank the aforementioned climate factors; see Table 5. Snow/ice and temperature were the only three factors and ranked as the highest priority (*n*: 23). All informants pointed out the relation between the two that without cold temperatures, there would not be any snow or ice. The one exception to the top three rankings was in a mountain region where one informant ranked temperature as most important, and then wind as heavy winds could be directly dangerous, and snow after that; see Table 5. Furthermore, three informants said that the other factors, precipitation, visibility, and wind, did not matter at all as they could adapt to any of these types of weather.

**Table 5:** Climate and weather priority order for dogsledders in Arctic Sweden

Priority order	Frequency (*n*)	Percentage (%)
1 and 2 temperature/snow, the rest does not matter	3	13.04%
1 and 2 temperature/snow, 2 precipitation, 3 wind, 4 visibility	12	52.17%
1 and 2 temperature/snow, 2 wind, 3 precipitation, 4 visibility	3	13.04%
1 and 2 temperature/snow, 2 visibility, 3 wind, 4 precipitation	2	8.69%
1 temperature, 2 wind, 3 snow, 4 precipitation, 5 visibility	1	4.34%

Snow on the ground was considered dependent on cold temperatures resulting in the informants ranking them as the same; both thresholds always taking the highest and second highest ranking, except for (*n*: 1)

Lastly, the informants were asked if they experienced an increase in extreme weather events and if so, what type; see Table 6. The most common answer was storms (*n*: 15) followed by an increase in forest fires (*n*: 6) and more variation/rapid changes in the weather (*n*: 6). This corresponds with studies done by SMHI (2015) and The Swedish Civil Contingencies Agency [MSB], which have invested in new resources to fight the increased risk and intensity of forest fires; these factors include heatwaves, storms, and thunderstorms (MSB 2022a, b). Worth noting is that some informants stated that they believed that forestry could be one contributing factor to the increase in storms and forest fires (*n*: 3).

**Table 6:** Experience of extreme climate and weather events for dogsledders in Arctic Sweden

Extreme climate or weather event	Frequency (*n*)	Percentage (%)
Storms	15	65.21%
Forest fires	6	26.08%
Variations/changes in weather	6	26.08%
Heatwaves	5	21.74%
Heavy rain	5	21.74%
Floods	5	21.74%
Heavy snowfall	4	17.39%
More extreme weather	3	13.04%
Thunderstorms	2	8.69%
Warmer seawater	1	4.34%

### Future climatic thresholds for dogsledding recreation and tourism in Northern Sweden

**Fig. 3:**
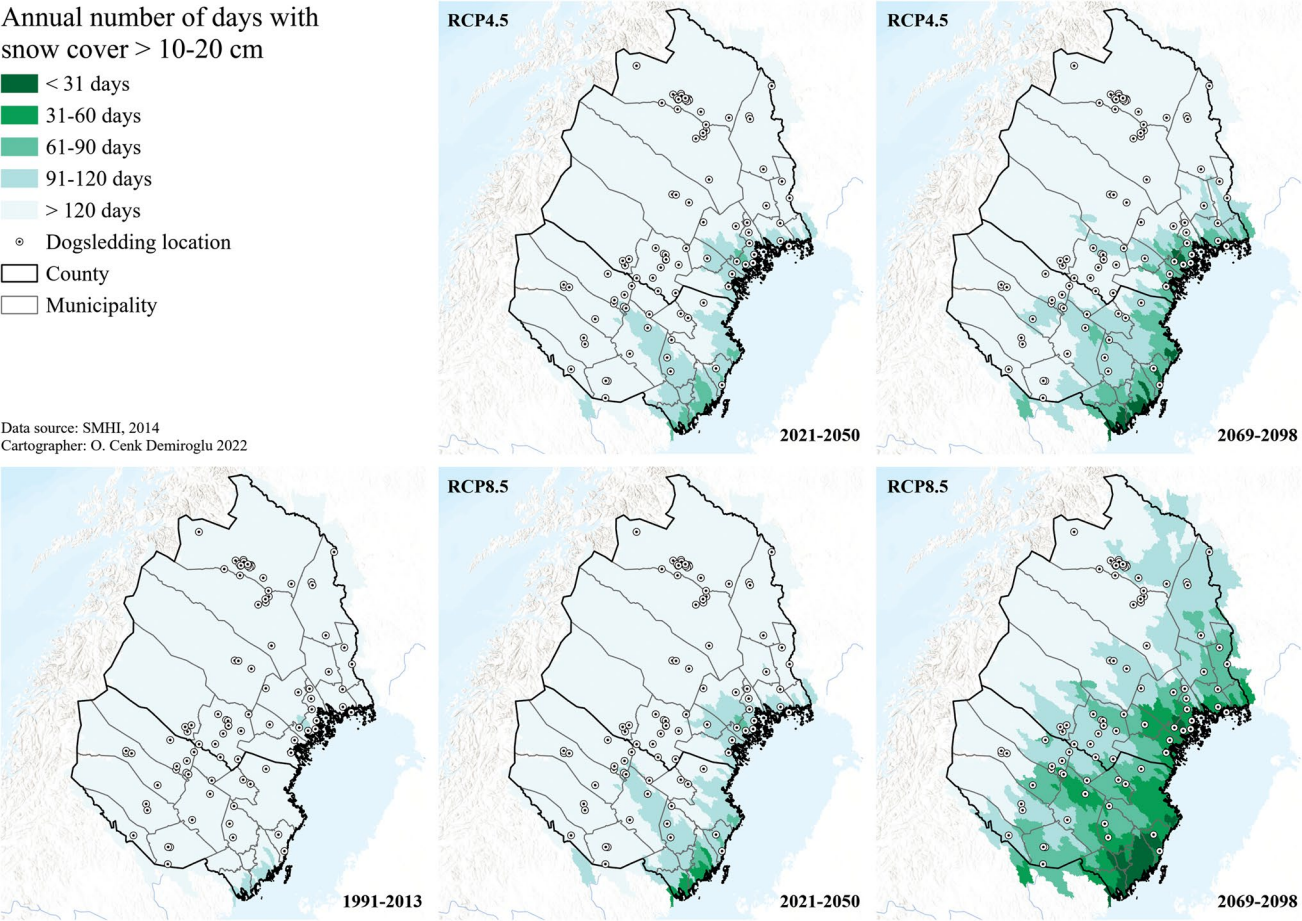
Future snow days for dogsledding climate in Arctic Sweden

The climate and weather thresholds that emerged from the interviews were compared to climate projections (SMHI 2014) to determine the future possibilities for dogsledding recreation and tourism in the region. One of the most critical thresholds, i.e., sufficient snow cover, was visualized based on the hydrological index “number of days with snow cover with a minimum water content of 20 mm” (SMHI 2015) since such water content threshold is equivalent to a snow depth of 10 cm with a wind-packed fresh snow density of 200 kg/m^3^ (SMHI 2021a). The historical (1991–2013) projections were consistent with the conventional snow cover duration of more than 4 months of dogsledding, but the season was further projected to be reduced below 3–4 months along the coastal areas, and even in the southern inland, as soon as 2021–2050, regardless of the GHG trajectory (Fig. 3). By the end of the century (2069–2098), such shortening of the season extends even further into inland and higher latitudes, especially at the expense of lack of mitigation (RCP8.5), with some coastal businesses having their seasons reduced below 1 month. Moreover, it should be noted that these findings do not necessarily ensure consecutive snow reliable days to make up a concrete season. Nor are their interannual timings certain, preventing us from understanding the risks of missing peak demand times such as Christmas, Easter, and school holidays. Hence, the impact findings may be interpreted towards an even more negative future.

**Fig. 4:**
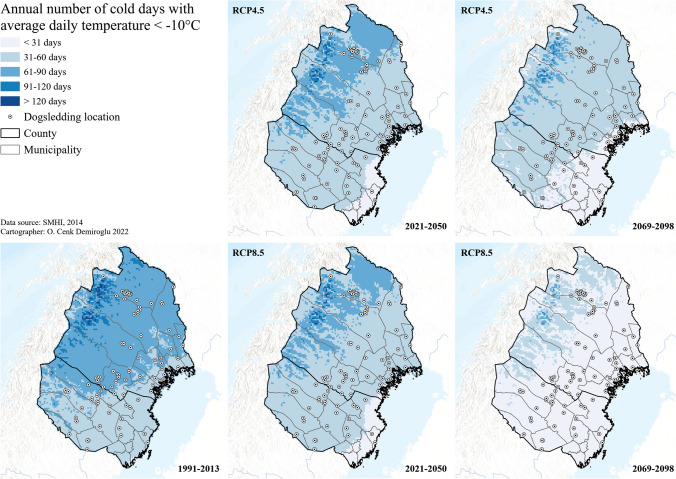
Future cold days for dogsledding climate in Arctic Sweden

In addition to the snow index, the two meteorological indices on the number of days with average daily temperatures below − 10 °C and above 20 °C were also utilized to understand the climatic futures of dogsledding. The first one can be used as a proxy for the dogsledding ice layer, as lake/river/sea ice formation is dependent on freezing temperatures, besides the turbulence factor. In the past, such cold temperatures lasted for 2 months in the coastal and southern dogsledding zones, and up to 3 months in the more northerly, inland areas (Fig. 4). In the near future (2021–2050), these conditions could shortened by a month, except around the northernmost and the most inland municipalities of Gällivare and Kiruna, which also lose such advantage in the further future and even under the mitigation scenario (RCP4.5). By the end of the century, and under the worst scenario (RCP8.5), such freezing temperatures can only last for less than a month in almost the entire present dogsledding geography of Arctic Sweden.

**Fig. 5:**
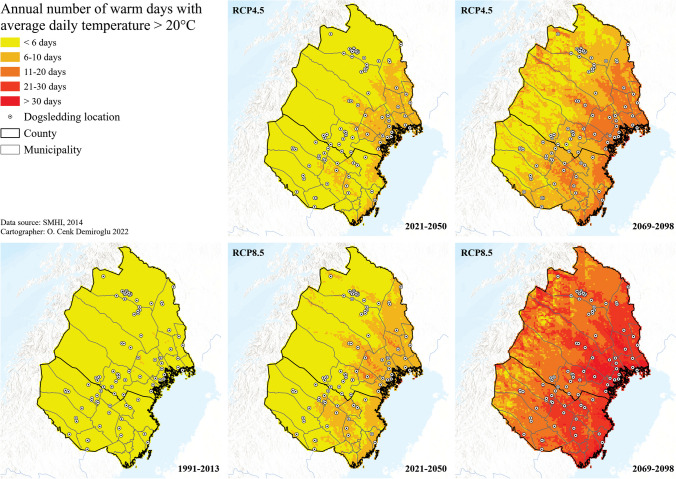
Future warm days for dogsledding climate in Arctic Sweden

The warm days’ index provided an understanding of the warm temperature thresholds for the dogs, especially during the summer. Accordingly, a doubling of such weather was projected for the coastal and southern municipalities soon (2021–2050) (Fig. 5). Warming significantly intensifies and expands for the far future, where the RCP8.5 scenario indicates the annual number of warm days to reach 11–20 days in the inland and 1 month or more in the coastal and the southern areas. Higher temperatures combined with low soil moisture could lead to more fire risks. Indeed, SMHI (2014) projects an increasing length of the fire risk season in Sweden, which becomes more prominent for the northern parts by the end of the century.

**Fig. 6:**
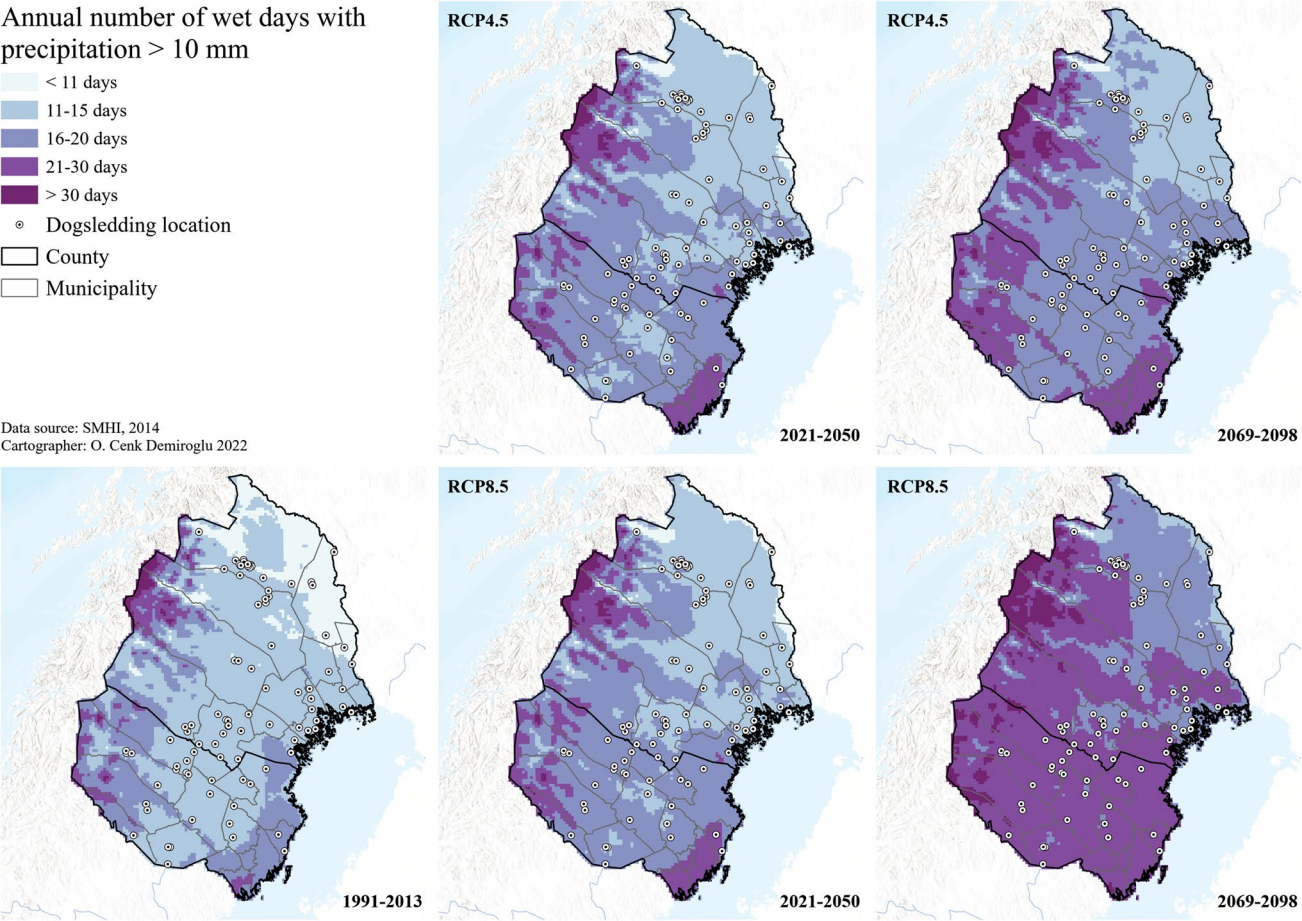
Future days with precipitation for dogsledding climate in Arctic Sweden

Regarding other extreme types of climate and weather, the index on the number of days with precipitation over 10 mm was used as a proxy for rainfall effects. Here, almost all regions, including even the driest northernmost areas, are projected for increases soon (2021–2050) whereas the far future (2069–2098) becomes even wetter with almost all dogsledding locations having annual precipitation days of 3 to 4 weeks under the RCP8.5 scenario (Fig. 6). It should, however, be noted that this index does not distinguish the type of precipitation (snow vs rain) and timing of the precipitation (dogsledding season or not). As for other extremes, SMHI projects insignificant or reduced risks of flooding for Västerbotten (Vindel River) and Norrbotten (Torneå River), respectively, contrasting the increases for the rest of the country—the reasons being the reduced snow amounts in the North and the increasing extreme rainfall in the South (Sjökvist et al. 2015). The linkage and the future of storm events to climate change, on the other hand, are still debatable, according to SMHI (2021b). This also applies to any storm surge risks for the coastal regions, which are also relatively resilient against the ongoing sea level rise being compensated by the glacial isostatic adjustment (Hieronymus and Kalén 2020).

## Concluding discussion

Our sample of 24% (*n*: 23) of a total of 97 dogsled businesses includes informants from across the region, including various municipalities in the inland, coastal, and mountain zones. As our results show, the informants had similar climate and weather thresholds regardless of their location. However, it should be noted that the businesses that chose not to participate in the study due to lack of time or interest may have other climate and weather thresholds. Despite that, the results from the informants in the study all highlighted the same three significant thresholds for snow depth, ice thickness, and warm and cold temperatures. The informants specified the critical minimum thresholds of packed snow trails and ice thicknesses of 10–20 cm for dogsledding activities, with some exceptions, where some informants wanted 30–40 cm of packed snow for uneven trails (*n*: 2) and 20–30 cm of ice thickness for safety (*n*: 2). These thresholds are similar to previous studies in Finland, with a snow cover of 11–20 cm and ice thickness of 15–20 cm (Tervo 2008) and in Greenland, 20 cm of snow (Schrot et al. 2019). Yet, this study contributes further by specifying that these measurements are for packed snow in the trails and not natural depth. The results also support the statement by Tiller and Richard (2018) that if the snow disappears, dogsledding using sledges will no longer be possible. The warm temperature threshold was a maximum of 15 °C (*n*: 21) with two exceptions 5 °C (*n*: 1) and 0 °C (*n*: 1) and was set for the dogs’ safety to avoid being overheated and injured. Three factors were found to affect the maximum temperature: humidity, snow on the ground, and access to bodies of water. Humidity lowers the threshold, as more humid weather is experienced as warmer in the summer. Snow and bodies of water are means to cool down the dogs resulting in higher thresholds. No correlations between dog breeds and maximum threshold temperature were found. These findings correspond with Äijälä (2022) results that dogsledders in Finland would not train their dogs above 15 °C, and Tervo (2008) stated that plus degrees were the most critical consequence for dogsledding and other winter activities. Cold temperature thresholds for dogsledding have not been studied before, and it was found that these thresholds were determined to meet the visitor’s preferences and were not considered a problem for dogsledders themselves or the dogs. Twelve informants had no specific cold limit, and 11 informants had a cold limit between − 25 and − 41 °C based on previous visitors’ experience. Informants from both groups let visitors determine if it was too cold, and if children participate, it was up to them to decide what was too cold.

Rain was considered problematic for the informants as it could result in tour cancellations, as already experienced in the case study area in Greenland (Schrot et al. 2019) and was seen as the most crucial threshold along with warm temperatures (Tervo 2008). It was also found that snow could cause a problem in connection with the wind. Strong winds could lead to snowdrifts over the trails, trees falling, tipping of sledges, and/or having a negative effect on visitors’ experiences (*n*: 8). One informant in the mountain region specified that wind by itself could be potentially dangerous demonstrating that climate and weather thresholds vary between places. The only non-crucial climate and weather threshold that was found was visibility (fog, mist, cloudiness). However, dogsledders said that most visitors preferred cloud-free experiences. Last but least, other critical extreme events to threaten the future of dogsledding tourism were stated as personal experiences of the informants in their respective locations. These were identified as heatwaves, storms, thunderstorms, and forest fires. The informants’ own experiences are similar to the findings of The Swedish Civil Contingencies Agency [MSB] 2022a, b), and The Swedish Meteorological and Hydrological Institute [SMHI] (2015), whose studies show an increased number and intensity of forest fires. Some informants in this study had experienced more floods from different rivers and lakes, yet SMHI’s projection for the future does project an insignificant or reduced risk of flooding for Västerbotten (Vindel River) and Norrbotten (Torneå River), respectively, in the future (Sjökvist et al. 2015). The climate projections revealed a shortening of the season due to reduced snow and ice cover for the southern and coastal locations. According to these projections, this would occur in the near future (2021–2050), and an overall worsening scenario for the far future (2069–2098) in the absence of global mitigation measures (RCP8.5), with few exceptions in the mountain regions to the west. A similar pattern was also found for the warm day frequencies in the future (2069–2098), implying safety risks for the dogs and increased fire risks. Increasing precipitation is another future trend that may continue to have negative consequences such as tour cancellations. It was found that the coastal region would be the first to be affected by climate change and would see the most drastic changes in the worst-case projections by SMHI. These results can be compared to the findings of Brouder and Lundmark (2011) and may explain why their informants in the coastal regions experienced greater shifts in their surroundings due to climate change compared to their informants in the inland regions.

This study aimed to fill the descriptive research gap that exists about the dependency on the physical environment, climate, and weather for dogsledding activities. This was achieved by assessing the impacts of climate change on dogsledding recreation and tourism in Northern Sweden based on expert information from dogsledders in the case study area and the climate projection data from SMHI (2014, 2015, 2021b). The results provide a first step in creating a climate index for dogsledding recreation and tourism. Winter tourism activities in many places have already experienced a lack of snow reliability and increased snow-making demand, which has led to suffering from economic damages and livelihood failure due to climate change (high confidence) (IPCC 2022). The types of indices found in this study are thus important to gain a better understanding of destinations’ livelihoods and their future adaption possibilities due to climate change as argued by Demiroglu et al. (2021).

Furthermore, the preliminary findings of the present study show that future studies should investigate adaptation strategies due to the changing environment among dogsledder and other nature-based activity tourism businesses. Dogsledders in this study were well aware of the changing environment, and several of them had already started planning and/or working to adapt to the changing climate. Some findings resonated with product diversification ideas (change from dogsledding to all-terrain vehicles) in Norway (Tiller and Richards 2018) and temporal adjustments (from multi-day to single-day tours) in Greenland (Schrot et al. 2019). It also revisits the relocation intention, especially among the dogsledders located in less snow-secure areas of Northern Sweden (Brouder and Lundmark 2011). These findings were however outside the scope of this study, and more data is needed before drawing any conclusion. Yet, it highlights the need for more research to understand the climate vulnerability/resilience and adaption capacity of dogsledding recreation and tourism geographies. This includes both business, product, and marketing adaptation as destinations in this region and others are in many cases dependent on these types of nature-based activity businesses (Margaryan 2018). Such research would contribute to a better understanding of the importance of nature-based activities for particular destinations, such as in the case of Rovaniemi (Finland) and Santa Claus tourism (Tervo-Kankare et al. 2013), and the consistency with the wider ongoing “Arctification” trend in the region (Marjavaara et al. 2022). Furthermore, it calls for studies about imaginary projections of places and products that are dependent on the environment, climate, and weather, i.e., the attributes that tourism and other types of stakeholders use to promote to convey the desired image. The imaginary projections used in marketing have a great influence on how a place is perceived by others (Hunt 1975), and natural resources are an essential part of tourism branding strategies (Wall Reinius and Fredman 2007). For the dogsledders in this study, and for winter tourism in general in the region, snow, ice, cold, and other environment-related imaginaries are important attributes used for naming, branding, marketing, and promoting destinations, businesses, products, and services (see for example, Jacobsen 1997; Lucarelli and Heldt Cassel 2019; Marjavaara et al. 2022; Viken and Granås, 2014). Places and products that are marketed using certain climate and weather attributes to promote specific imaginaries can otherwise become problematic if the natural environment changes and no longer matches what is being promoted (Hall 2008).

## Footnotes

1 The other breeds included Border Collies, Swedish Jämthund, various types of Lapphund, Spitz and Hounds, and mixes of all the mentioned breeds. The breeds that were only family dogs and not used for sledging were not included as these were not relevant to the study.
